# ZIF-8-Derived Multifunctional Triethylamine Sensor

**DOI:** 10.3390/s24165425

**Published:** 2024-08-22

**Authors:** Shuo Xiao, Zheng Jiao, Xuechun Yang

**Affiliations:** School of Environmental and Chemical Engineering, Shanghai University, Shanghai 200444, China; sxiao83@126.com (S.X.); xuechunyang@i.shu.edu.cn (X.Y.)

**Keywords:** ZIF-8, triethylamine, sensor

## Abstract

Triethylamine (TEA) is a typical volatile organic compound (VOC) widely present in air and water, produced in industrial production activities, with high toxicity and great harm. Fluorescence detection and resistive sensing are effective methods for detecting pollutants. Here, In-doped interpenetrating twin ZIF-8 and its annealed derivatives have been successfully designed and prepared as a multifunctional TEA sensor. On the one hand, ZIF-8-In exhibits excellent fluorescence emission enhancement at 450 nm in a dose-dependent manner to TEA in water within the concentration range of 1–100 ppm, with a detection limit as low as 1 ppm. On the other hand, the annealed ZIF-8-In derivative is ZnO/In_2_O_3_ with a porous hierarchical structure, which is a perfect sensitive material for manufacturing gas sensors. Within the concentration range of 1–100 ppm, the ZnO/In_2_O_3_ gas sensor has a high response for 100 ppm TEA, reaching 107.7 (Ra/Rg), and can detect TEA gas as low as 1 ppm. Furthermore, the response of ZnO/In_2_O_3_ sensors to TEA is at least 10 times that of the other four VOC gases, demonstrating excellent gas selectivity. This multifunctional sensor can adapt to complex detection situations, demonstrating good application prospects.

## 1. Introduction

The world is facing severe air pollution, due to rapid industrialization and urbanization, emitting a large amount of harmful and polluting gases, such as volatile organic compound gas (VOC), NO, NO_2_, etc. [1,2,3,4,5,6]. These toxic gases will seriously affect the respiratory system, and even lead to the abnormal composition of pulmonary surfactant, resulting in lung damage. Triethylamine (TEA) is a common gas pollutant secreted from industrial casting, the fine chemical industries, and so on. Decaying fish also can release TEA gas; hence, the concentration of TEA serves as one of the indicators for assessing food spoilage. TEA, as a toxic and harmful VOC, can cause strong irritation to the eyes, skin, and respiratory tract, and may lead to pulmonary edema and even death [7]. The National Institute for Occupational Safety and Health (NIOSH) in the United States recommends a TEA concentration of 10 ppm in the workplace, while the American Conference of Government Industrial Hygienists (ACGIH) recommends a concentration of 1 ppm [8,9]. Therefore, many countries have identified TEA as a key monitoring target for industrial waste gas.

Metal organic frameworks (MOFs) are widely used in various applications due to their special design, unique pore size, adjustable structure, and large specific surface area, including molecular storage/separation [10], catalysis [11], luminescence-based chemical/biological sensing [12], and so on. MOF-based sensors stand out in the following aspects: first of all, the pore structure of MOFs provides a large specific surface area and many rich potential active sites, which are beneficial for improving their interaction efficiency with target molecules and facilitating rapid detection. Secondly, the pore size/geometric shape and physicochemical environment of customized MOFs provide selective adsorption for certain target molecules, leading to higher selectivity and sensitivity. Thirdly, the reversible absorption and release of target molecules by MOFs make MOF-based sensors highly renewable. Finally, the good thermal and chemical stability of most MOFs ensures the long lifespan of MOF-based sensors [13,14,15]. Among them, a zinc-imidazole framework 8 (ZIF-8), also known as zeolite imidazole salt framework (ZIFs), is widely popular due to its unique structure and physicochemical properties. For example, Li et al. proposed a surface-modified ZIF-8 cascaded tapered fiber optic sensor. The sensor has a good response to ethanol within the concentration range of 0–140 ppm at room temperature, with a sensitivity of 0.1411 nm/ppm. This sensor has high sensitivity and a good reversibility and online detection capability, making it a convenient tool for monitoring ethanol leakage in industrial production [16]. Yang et al. synthesized ZIF-8 crystals doped with Cd. This new material exhibits high fluorescence excited by 380 nm, with a maximum emission wavelength of 444 nm. Furthermore, a sensor device based on a mixed matrix membrane was prepared using PDMS (polydimethylsiloxane) as the substrate for Cd-doped ZIF-8 crystals, resulting in a fluorescent sensing film with rapid selective response to a large number of potential interferences [17]. Xu et al. report a flexible sensor based on dual-emission ZIF-8 for the detection of antibiotics in water. The sensor can perform dual-emission ratio fluorescence sensing on furantoin and oxytetracycline, exhibiting sensitive detection for fluorescence burst and fluorescence enhancement, with detection limits of 0.012 μM and 8.9 nM, respectively [18]. Ren et al. reported a highly sensitive and selective NO_2_ gas sensor based on a porous ZnO nanocube derived from ZIF-8. The ultra-high sensitivity and selectivity are attributed to the unique structure after pyrolysis, which provides more exposed active sites and connected pores [19].

Based on the above inspiration, ZIF-8-In was designed and prepared, and its morphology and structure were analyzed by AEM and TEM. What is more, the crystal structure of ZIF-8-In was analyzed through XRD. Trace amounts of TEA in water were detected by testing the fluorescence emission intensity of ZIF-8-In in different concentrations of TEA aqueous solutions. Subsequently, annealed ZIF-8-In derivatives were used as gas sensors to detect TEA gas in the air. Both detection methods have ultra-sensitive responses to TEA in water and air; therefore, the specific detection mechanisms were analyzed in detail. This multifunctional TEA gas sensor can adapt to complex detection environments to ensure accurate monitoring of TEA content in air and water.

## 2. Materials and Methods

### 2.1. Materials

The precursor materials, Zn(CH_3_COO)_2_ (99.99%), Hexadecyl trimethyl ammonium bromide (CTAB, 99%), In(C_2_H_3_O_2_)_3_ (99.99%), 2-Methylimidazole (2-MeIM, 98.0%), CH_3_CH_2_OH (95%), and C_2_H_6_O (99.8%) were purchased from Aladdin Reagent Co., Ltd., Shanghai, China. All chemical reagents involved in the experiments are of analytical purity and were used directly without further purification. Ceramic substrate was purchased from Sensor Brainwave Co., Ltd., Suzhou, China, Circuit boards and the bases of the gas sensors were purchased from Winsen Electronic Technology Co., Ltd. Zhengzhou, China. 

### 2.2. Preparation of ZIF-8-In and ZnO/In_2_O_3_

Firstly, 1.5 mmol Zn(CH_3_COO)_2_ and 0.35 mmol Hexadecyl trimethyl ammonium bromide were dissolved in 50 mL deionized water to obtain solution A. Next, 30 mmol 2-Methylimidazole was dissolved in 30 mL deionized water to obtain solution B. Solution A was poured into solution B and stirred for half an hour to obtain solution C. Then, solution C was transferred to a 100 mL hydrothermal reactor and subjected to hydrothermal reaction at 120 °C for 24 h. Subsequently, methanol and ethanol were used alternately to clean the product 3–5 times and dried at 80 °C for 8 h to obtain ZIF-8. Afterwards, 0.08 g In(C_2_H_3_O_2_)_3_ was dissolved in a mixed solvent of 1:3 ethylene glycol and ethanol, and stirred for 40 min. Then, 0.15 g of ZIF-8 was added and stirred to form a uniform solution. Finally, the solution underwent hydrothermal reaction at 160 °C for 4 h. The obtained product was washed twice with ethanol and deionized water to obtain ZIF-8-In. Three grams of ZIF-8-In was annealed at 500 °C for 3 h to obtain ZnO/In_2_O_3_. The blank control group consists of ZIF-8 and ZnO obtained by annealing ZIF-8.

### 2.3. Characterization

A scanning electron microscope (SEM, Regulus 8100, Hitachi, Japan) was used to observe the morphology structure of ZIF-8-In. The nanoscale microstructure of the samples was observed using a high-resolution transmission electron microscope (HRTEM, JEM-2100F, JEOL, Japan). A quantity of 0.01 mg of the sample powder was dispersed in 5 mL of ethanol and sonicated for 5 min. Capillary tubes were used to take a drop of sample and dropwise it on the smooth surface of a single crystal silicon wafer for SEM measurement, or dropwise it on a 300 mesh copper mesh for TEM testing. The adsorption–desorption isotherms of nitrogen measurement were handled on an automated surface analyzer (Quadrasord SI, Quantachrome, Tallahassee, FL, USA). The crystalline structures of ZIF-8-In and ZnO/In_2_O_3_ were analyzed using X-ray diffraction (XRD, D/MAX2200V PC, Cu Kα, k = 1.5406 A, Rigaku, Japan), with a scanning range from 5 to 80°. X-ray photoelectron spectroscopy (XPS) was performed using a K*α* spectrometer (Thermo Scientific K-Alpha+, Waltham, MA, USA) with an Al K*α* X-ray source. The photo-luminescence (PL) spectra were obtained on a fluorescence spectrometer (FLS1000, Edinburgh, Britain). The sensing performance of the ZnO/In_2_O_3_ gas sensor was tested using a gas sensor measurement system (WS-30A, Winsen Electronics Technology Co., Ltd., Zhengzhou, China).

## 3. Results

**Figure 1:**
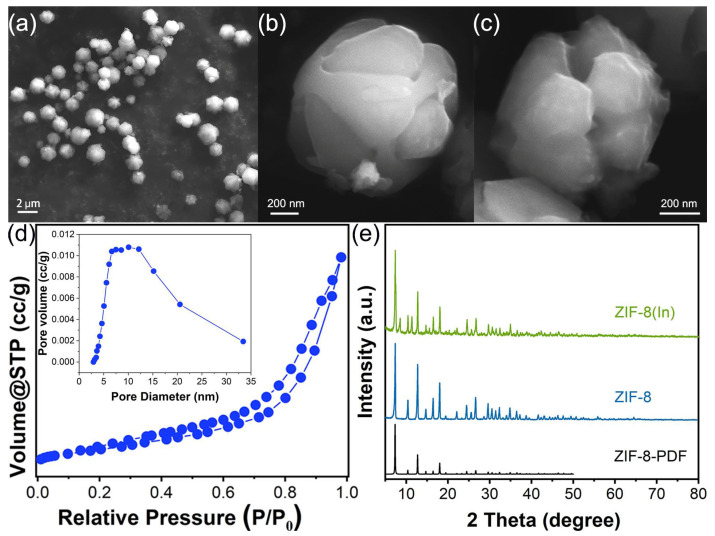
(**a**,**b**) The SEM images of ZIF-8, (**c**) the SEM image of ZIF-8-In, (**d**) the BET results of ZIF-8-In, (**e**) the XRD patterns of ZIF-8 and ZIF-8-In.

The ZIF-8 and ZIF-8-In were successfully prepared and their morphology was evaluated using clear and high-quality SEM images. As shown in Figure 1a,b, the grain size of ZIF-8 is around 1–2 µm. ZIF-8 has a complete particle size and good monodispersity. After ion exchange with In, the morphology of ZIF-8-In had not changed compared with ZIF-8, indicating that the ion exchange process did not cause a collapse or change in crystal structure (Figure 1c). In Figure 1d, the BET results show that ZIF-8-In has abundant mesopores, with pore sizes distributed between 6 and 12 nm. The adsorption–desorption curve of ZIF-8-In does not have a clear saturated adsorption platform, indicating an irregular pore structure and a wide range in pore size distribution. Compared with the published XRD result of ZIF-8, both ZIF-8 and ZIF-8-In are pure phases [20]. The strong characteristic diffraction peaks of ZIF-8-In at 2θ = 7.4°, 10.4°, 12.7°, 14.7°, 16.4°, 18.0°, and 22.1° correspond to the (011), (002), (112), (022), (013), (222), and (114) crystal planes of ZIF-8, respectively [21]. 

**Figure 2:**
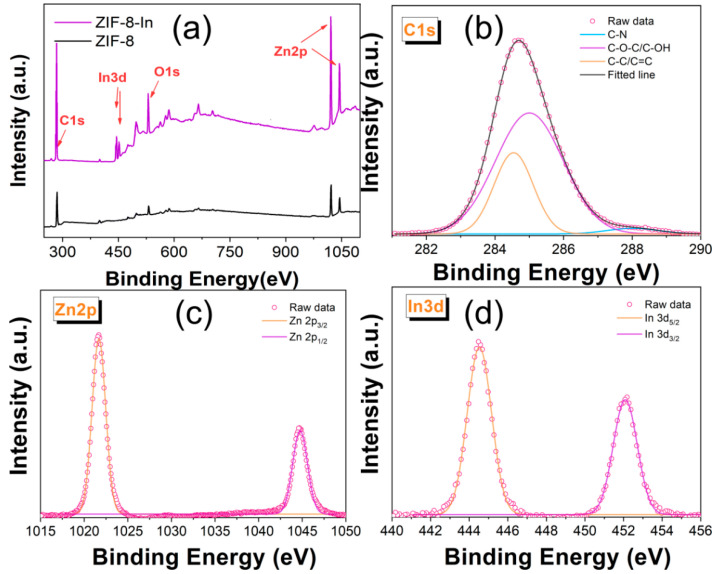
(**a**) The survey spectra of ZIF-8 and ZIF-8-In, the high-resolution (**b**) C1s, (**c**) Zn2p, and (**d**) In3d spectra of ZIF-8-In.

Furthermore, the surface structure of ZIF-8 was analyzed in detail through XPS results. As shown in Figure 2a, ZIF-8 mainly contains three elements: C, O, and Zn. After doping with In, the strong In 3d peaks appeared in the survey spectrum of ZIF-8-In. The high-resolution XPS spectra of C1s show that ZIF-8-In contains C-C/C=C (284.5 eV), C-O-C/C-OH (285.1 eV), and C-N (288.1 eV) (Figure 2b). Rich functional groups are effective active sites for detecting TEA. Figure 2c shows two symmetrical peaks in the Zn2p spectrum of ZIF-8-In, with peaks at 1022.1 eV corresponding to Zn2p_3/2_ and 1044.6 eV corresponding to Zn2p_1/2_, indicating that the zinc ion is a single type of Zn^2+^. The high-resolution In3d spectrum shows In3d_5/2_ peak at 444.6 eV and In3d_3/2_ peak at 452 eV, respectively (Figure 2d) [22]. 

**Figure 3:**
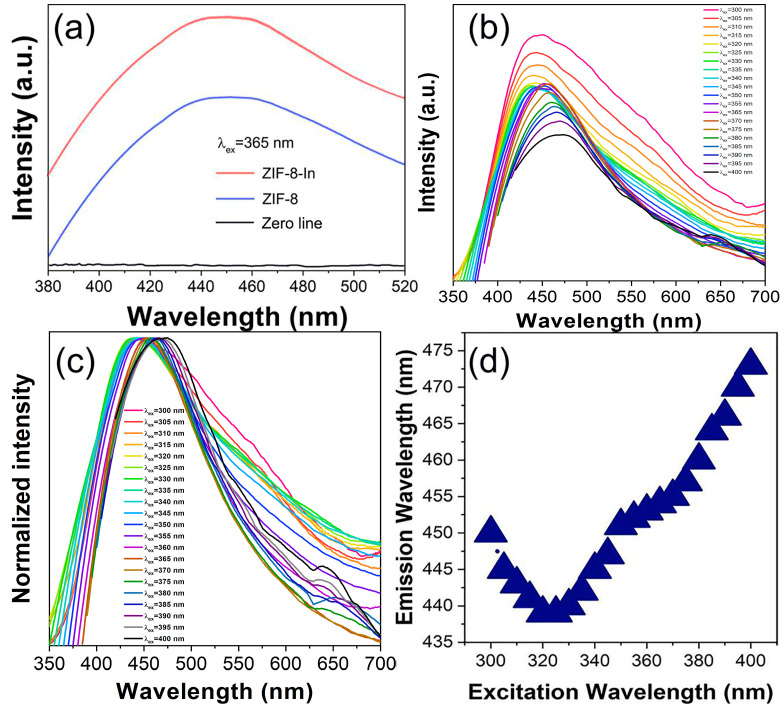
(**a**) The emission spectra of ZIF-8 and ZIF-8-In excited by 365 nm, (**b**) the emission spectra, and (**c**) normalized emission spectra of ZIF-8-In under different excitation wavelengths, (**d**) the trend of emission peak position of ZIF-8-In with the change in excitation wavelength.

Indium doping can often effectively improve the photo-response ability of materials [23]. As shown in Figure 3a, compared to pure ZIF-8, after indium doping, the fluorescence emission intensity of ZIF-8-In is significantly enhanced, which is beneficial for the strong signal output of subsequent fluorescence detection processes. In Figure 3b, the fluorescence spectra show that ZIF-8-In exhibits strong and stable blue fluorescence emission at approximately 450 nm. With the continuous redshift of the excitation wavelength, the fluorescence emission intensity of ZIF-8-In first strengthens and then weakens. Through the normalization analysis, it can be observed that, as the excitation wavelength changes from 300 nm to 400 nm, the peak position of ZIF-8-In emission spectra also undergoes significant changes (Figure 3c). The fluorescence emission peak of ZIF-8-In shifts blue first and then red. The maximum emission intensity of ZIF-8-In is achieved under 365 nm excitation (Figure 3d). Therefore, 365 nm was chosen as the optimal excitation wavelength for subsequent fluorescence detection TEA experiments. Also, 365 nm is a commonly used UV wavelength, which is easy to implement and ensures the widespread practicality of ZIF-8-In fluorescence sensing.

**Figure 4:**
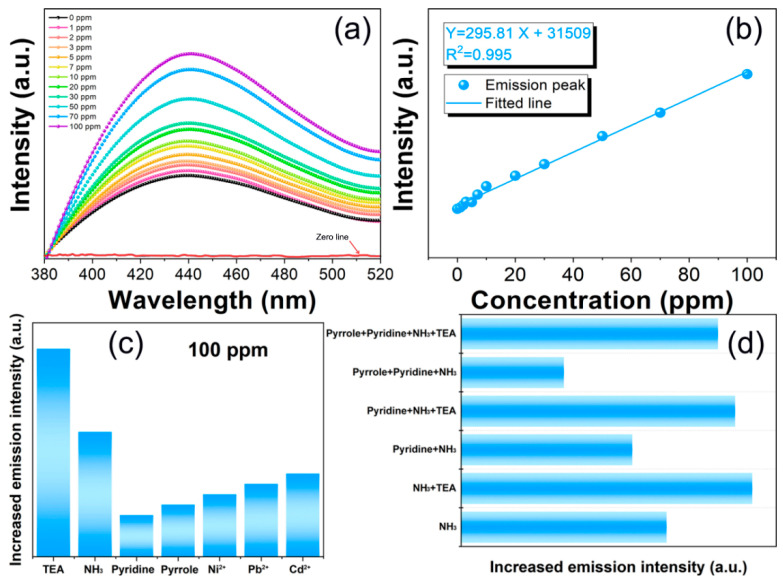
(**a**) The change in fluorescence intensity of ZIF-8-In with the addition of 1–100 ppm TEA, (**b**) relationships between the concentration of TEA and emission intensities of ZIF-8-In, the selectivity of ZIF-8-In for (**c**) 100 ppm single pollutants and for (**d**) 100 ppm mixed pollutants.

As shown in Figure 4a, the fluorescence detection experiment was conducted on the TEA content in water. With the increase in TEA content in the water, the emission peak intensity of ZIF-8-In significantly increases, while its peak position remains unchanged, exhibiting a typical dose-dependent fluorescence enhancement. The fluorescence-enhanced detection of ZIF-8-In is more conducive to the development of visual detection and real-time monitoring [23,24]. In Figure 4b, a functional relationship between the fluorescence emission intensity of ZIF-8-In and the TEA content in water has been established, which is beneficial for predicting TEA with unknown concentrations in water. The fluorescence detection limit of ZIF-8-In for TEA is 1 ppm, and the Chinese Ministry of Health has set the detection limit for TEA in water sources to 3 ppm. This means that the ZIF-8-In fluorescence sensor has good practical value and can meet the detection requirements for TEA concentration in water in production and daily life. According to the previous analysis, the surface of ZIF-8-In contains abundant functional groups. The -NH_2_ bond on TEA can form a large number of hydrogen bonds with C-N, C-O, -CONH-, etc. on ZIF-8-In. This weakly conjugated structure can greatly enhance the degree of electron cloud delocalization, increase the concentration of photo-generated electrons, and significantly enhance the fluorescence emission intensity of ZIF-8-In, achieving fluorescence-enhanced sensing. Furthermore, the selectivity of ZIF-8-In towards multiple single and mixed pollutants was tested (Figure 4c,d). Among various nitrogen-containing compounds, ZIF-8-In exhibits a higher response to TEA. In mixtures containing TEA, the response of ZIF-8-In is generally higher than that of mixtures without TEA.

**Figure 5:**
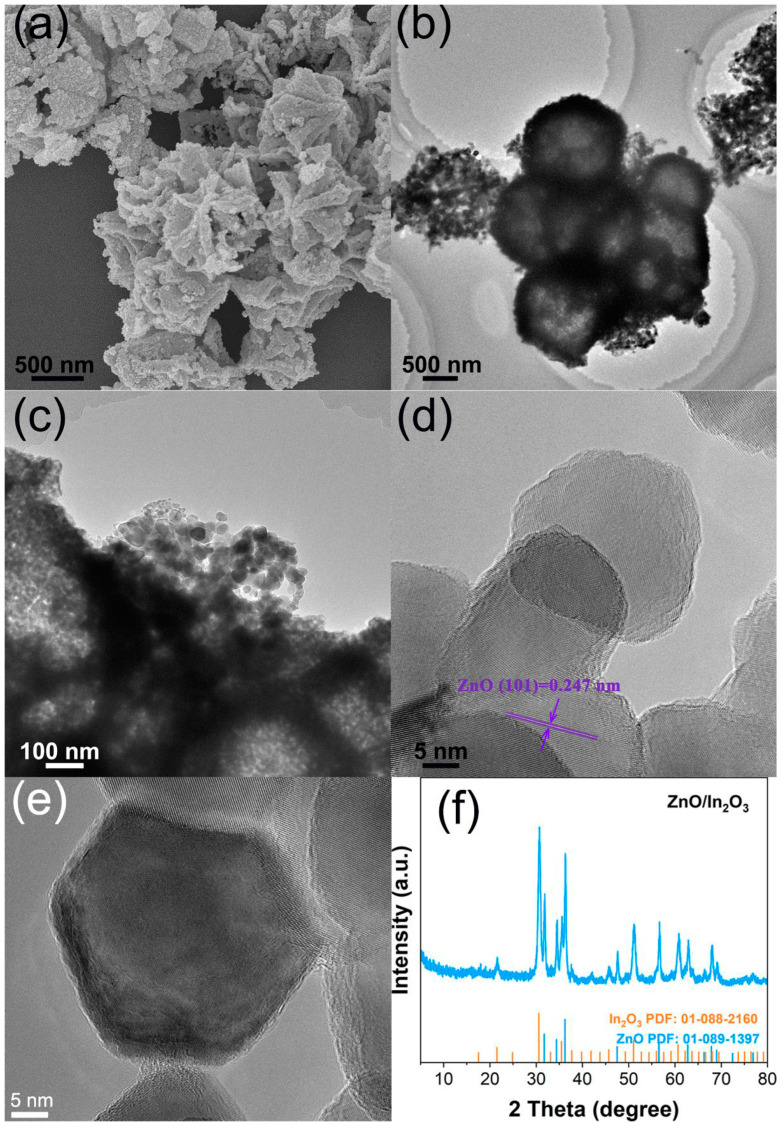
(**a**) The SEM image of ZnO/In_2_O_3_, (**b**–**e**) the TEM images of ZnO/In_2_O_3_, (**f**) the XRD result of ZnO/In_2_O_3_.

ZIF-8-In was annealed at 500 °C to obtain ZnO/In_2_O_3_ with a porous hierarchical structure. ZnO/In_2_O_3_ shown in Figure 5a retains the complete skeletal structure of ZIF-8-In. Unlike the flat and smooth surface of ZIF-8-In grains, the surface of ZnO/In_2_O_3_ grains is rough and uneven. The TEM images in Figure 5b,c clearly show that ZnO/In_2_O_3_ has a hollow structure and abundant pores. The abundant pores and increased specific surface area of ZnO/In_2_O_3_ enable effective gas adsorption, desorption, and transport, which is beneficial for highly sensitive and rapid detection. Figure 5d,e show that the smallest structural unit of ZnO/In_2_O_3_ is the nanosheet, with a diameter of approximately 30 nm. The nanosheets have clear lattice lines with a crystal plane spacing of 0.247 nm, corresponding to the (101) crystal plane of ZnO [25]. The XRD result shows the product of ZIF-8-In annealing is ZnO/In_2_O_3_.

**Figure 6:**
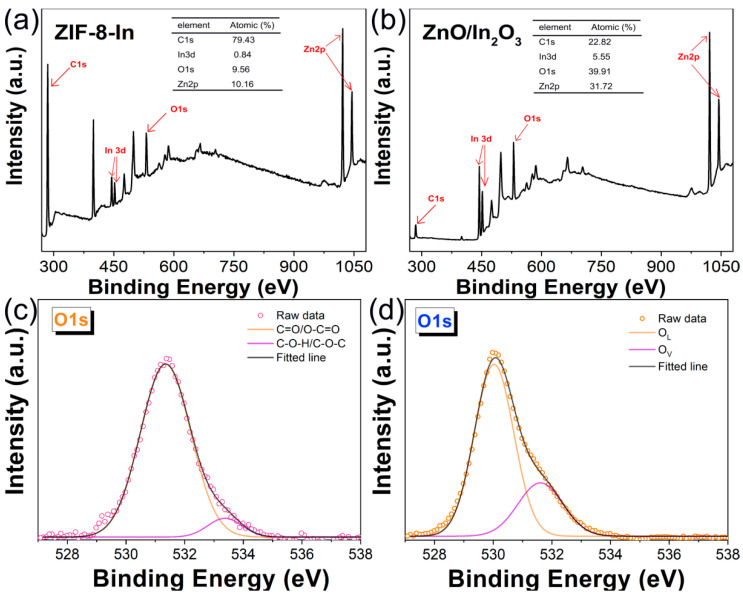
The survey XPS spectra of (**a**) ZIF-8-In and (**b**) ZnO/In_2_O_3_, the high-resolution O1s spectra of (**c**) ZIF-8-In and (**d**) ZnO/In_2_O_3_.

XPS semi-quantitative analysis showed the atomic percentages of ZIF-8-In and ZnO/In_2_O_3_, respectively (Figure 6a,b). Compared to ZIF-8-In, the content of In atoms in ZnO/In_2_O_3_ increases significantly after annealing, which is due to the loss of elements such as C and O. Figure 6c shows that ZIF-8-In has abundant functional groups, with peaks at 531.3 eV corresponding to C=O/O-C=O and 533.3 eV corresponding to C-O-H/C-O-C [26]. After annealing at 500 °C, ZnO/In_2_O_3_ was obtained from the high-temperature thermal oxidation of ZIF-8-In. As we know, lattice oxygen (O_L_) and vacancy oxygen (Ov) can usually be observed in the XPS high-resolution O1s spectra of semiconductor materials. O_L_ is very stable during gas-sensing reactions and does not contribute to gas-sensing performance. According to reports, Ov can provide active sites for gas adsorption and reaction on the surface of sensing materials, playing an indispensable role in high sensitivity [27]. The high-resolution O1s spectrum of ZnO/In_2_O_3_ in Figure 6d is not a Gaussian distribution, so it can be fitted as two peaks. The peak at 530.1 eV is O_L_, and the peak at 531.7 eV is Ov [28]. The rich content of Ov is beneficial for the efficient sensing process of the ZnO/In_2_O_3_ surface on TEA gas.

**Figure 7:**
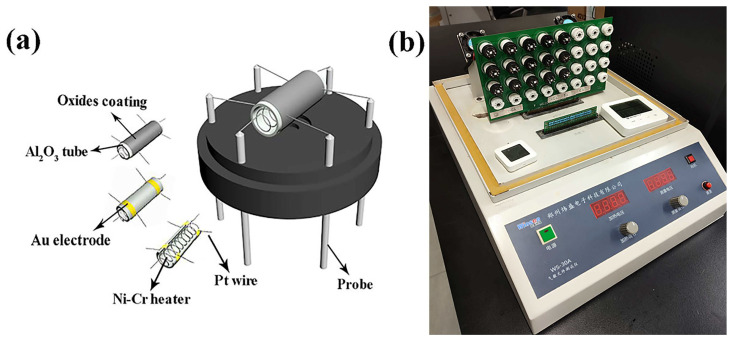
Manufacturing diagram of ZnO/In_2_O_3_ gas sensor, (**a**) structure diagram of gas sensor and (**b**) the photo of gas sensor measurement system.

ZnO/In_2_O_3_ with abundant pores and a large amount of Ov is an excellent sensitive material for making gas sensors. ZnO/In_2_O_3_ and terpineol were blended in a 3:1 weight ratio to form a slurry. The slurry was evenly applied on an Al_2_O_3_ hollow ceramic tube with a pair of Au electrodes and four Pt wires (inner diameter 0.8 mm, outer diameter 1.2 mm, length 4 mm). The Al_2_O_3_ tube coated with gas sensitive material was dried at 60 °C for 1 h to remove the terpineol. Then, a Ni-Cd heating wire with a resistance of 35 Ω was passed through the center of the Al_2_O_3_ tube. Four Pt wires and two heating wires were welded onto the components, as shown in Figure 7a. After aging at 300 °C for 3 days, the detection data were collected and analyzed using the WS-30A gas sensor measurement system (Wensen Electric Technology Co., Ltd. Zhengzhou, China) (Figure 7b). The working temperature was adjusted by changing the heating voltage, with a heating voltage range of 3.54 V to 5.29 V. In this work, the response value (S) of the ZnO/In_2_O_3_ gas sensor can be defined as S=Ra/Rg, where Ra and Rg refer to the measured resistance values in air and target gas under steady state, respectively. In addition, response time and recovery time refer to the time when the sensor resistance reaches a 90% equilibrium state during the gas injection and release processes, respectively [29].

**Figure 8:**
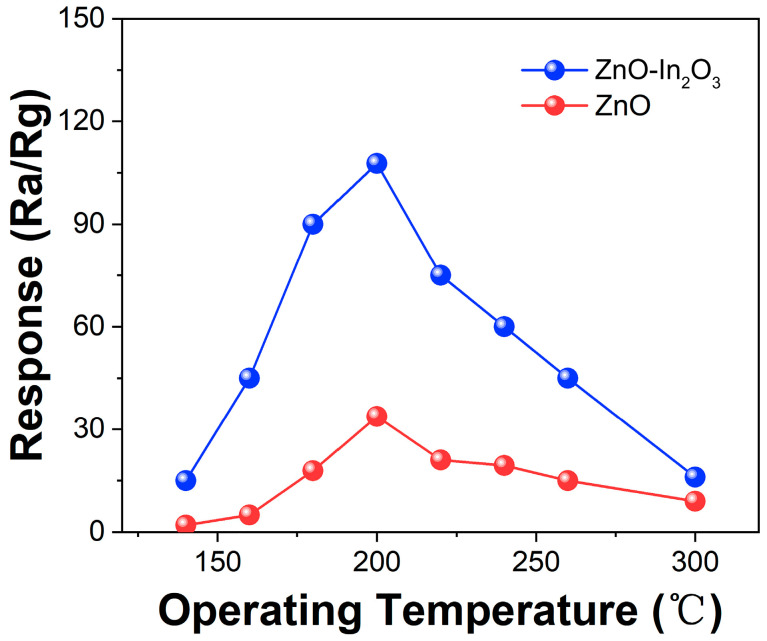
The gas response of the ZnO and ZnO/In_2_O_3_ sensors towards 100 ppm TEA at different operating temperatures.

As we all know, the operating temperature is an important index to show the gas sensor performance [30]. Figure 8 illustrates the correlation between the operating temperatures and the response of the gas sensor. The ZnO and ZnO/In_2_O_3_ sensors respond to TEA at 140–300 °C. In the range of 140–200 °C, the response signal of the ZnO/In_2_O_3_ gas sensor gradually increased with the increase in temperature until reaching a maximum response value of 107.7 at 200 °C, while the maximum response of ZnO to TEA was only 33.8 (200 °C). The TEA molecule will obtain enough energy to overcome the potential barrier of activation energy on the surface of ZnO/In_2_O_3_ at 200 °C, conducting effectively the redox reaction between the O^−^ and TEA, and reaching a high response. Subsequently, when the temperature continues to rise from 200 °C to 300 °C, the response signal of ZnO/In_2_O_3_ gas sensor began to weaken. The response curve of the sensor shows a pyramid shape with the change in temperature. The reason is that when the operating temperature is relatively low, TEA is not very active and does not have enough activation energy to react with the oxygen species adsorbed on the surface of the material, resulting in a low gas response. With the increase in working temperature, the adsorbed oxygen on the surface of the material increases, and the gas molecules also obtain enough energy to overcome the activation energy barrier, so the response also increases. However, when the operating temperature exceeds the optimal reaction temperature, the desorption of gas molecules may dominate, making the sensitivity decrease [31]. 

**Figure 9:**
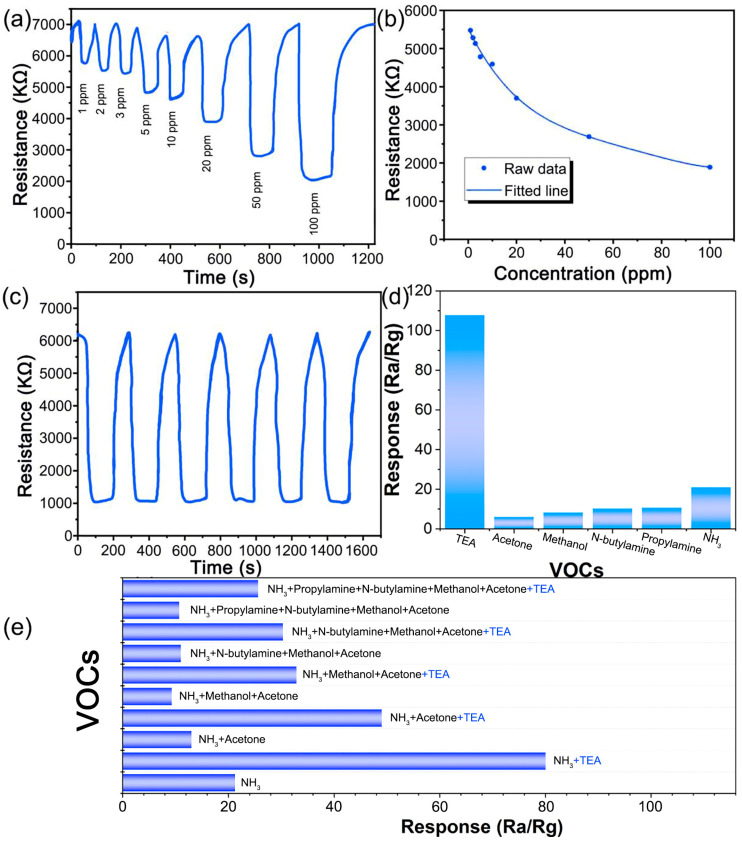
(**a**) The response of the ZnO/In_2_O_3_ sensor to 1–100 ppm TEA at 200 °C, (**b**) the relationships between TEA concentration and response of ZnO/In_2_O_3_, (**c**) the response repeatability curve of ZnO/In_2_O_3_ sensor to 100 ppm TEA at 200 °C, (**d**) the response of ZnO/In_2_O_3_ sensor to 100 ppm different VOCs at 200 °C, (**e**) the response of ZnO/In_2_O_3_ sensor to 100 ppm mixed VOCs at 200 °C.

The response of the ZnO/In_2_O_3_ gas sensor to TEA was systematically studied. The typical dynamic response and recovery curves of ZnO/In_2_O_3_ gas sensor for different TEA concentrations (1–100 ppm) are shown in Figure 9a. It can be observed that the response of the sensor steadily increases with the increase in TEA concentration. Even for TEA gas as low as 1 ppm, the ZnO/In_2_O_3_ sensor still exhibits a good response, indicating that it can be used for detecting trace amounts of TEA gas in air. The European Commission and the American Conference of Government Industrial Hygienists (ACGIH) both recommend setting the exposure threshold for TEA in the air to 1 ppm [32]. This means that the ZnO/In_2_O_3_ sensor has the ability to meet the detection needs in production and daily life, and has good practical value. The functional relationship between the response value of the sensor and the TEA concentration is shown in Figure 9b. The good fitting results indicate that the ZnO/In_2_O_3_ gas sensor can detect TEA gas in a wide concentration range of 1–100 ppm and can be used for quantitative detection of TEA. The cyclic response capability is also a key parameter of gas sensors, reflecting the stability of gas sensors [33]. In Figure 9c, the ZnO/In_2_O_3_ sensor undergoes six consecutive cyclic tests on 100 ppm TEA. The dynamic response and recovery curve is so stable and repeatable that the resistance can recover to its initial state within six cycles. Selectivity is another important characteristic of gas sensors. As shown in Figure 9d, the Ra/Rg values of the ZnO/In_2_O_3_ sensor for 100 ppm VOCs at 200 °C are as follows: TEA is 107.7, acetone is 6, methanol is 8.2, n-butylamine is 10.1, propylamine is 10, and NH_3_ is 20.8, respectively. Among them, the ZnO/In_2_O_3_ sensor has excellent selectivity for TEA, which is 18.0 times, 13.1 times, 10.7 times, 10.8 times, and 5.2 times that of acetone, methanol, n-butylamine, propylamine, and NH_3_, respectively. In order to further verify the selectivity of the ZnO/In_2_O_3_ gas sensor, several common VOC gases were mixed and configured into 100 ppm mixture gases with the same content. As shown in Figure 9e, the response of ZnO/In_2_O_3_ to mixed gases containing TEA is generally higher than that to mixed gases without TEA, demonstrating excellent selectivity towards TEA.

Finally, the detailed sensing process is explained by the following chemical equations:

O_2(ads)_ + 2e^−^ → 2O^−^_(ads)_ (1)

(C_2_H_5_)_3_N → NH_3_ + 3C_2_H_4_ (2)

2NH_3_ + 7O^−^_(ads)_ → 2NO_2_ + 3H_2_O + 7e^−^ (3)

C_2_H_4_ + 6O^−^_(ads)_ → 2CO_2_ + 2H_2_O + 6e^−^ (4)

In short, there is the oxidation process of TEA:

2(C_2_H_5_)_3_N + 43O^−^_(ads)_ → 12CO_2_ + 2NO_2_ + 15H_2_O + 43e^−^ (5)

Due to the electron affinity of oxygen molecules being greater than the work function of ZnO/In_2_O_3_, when the gas sensor is exposed to air, O_2_ molecules will be adsorbed on the sensor surface and thermally excited electrons will be removed from the conduction band to form a more active adsorbed oxygen species O^−^ (Equation (1)) [34]. Meanwhile, due to the loss of electrons, the resistance of the ZnO/In_2_O_3_ sensor will increase. Subsequently, when the ZnO/In_2_O_3_ sensor is exposed to TEA gas, TEA molecules will adsorb on the sensor surface and decompose into NH_3_ and C_2_H_4_ (Equation (2)). NH_3_ and C_2_H_4_ will be oxidized by O^−^ to NO_2_, CO_2_, and H_2_O (Equations (3) and (4)). Meanwhile, chemical reactions release free electrons back into the conduction band, resulting in a decrease in resistance.

## 4. Conclusions

In summary, ZIF-8-In was synthesized through a simple hydrothermal method. As a fluorescent sensor, ZIF-8-In can sensitively detect TEA in water with a detection limit as low as 1 ppm. Its weak conjugated structure can greatly increase the degree of electron cloud delocalization, increase the concentration of photo-generated electrons, and thus significantly enhance the fluorescence emission intensity of ZIF-8-In. The fluorescence-enhanced detection of ZIF-8-In is more conducive to the development of visual detection and real-time monitoring. Afterwards, simple annealing was performed to obtain the ZnO/In_2_O_3_ gas sensor. The gas sensor can sensitively detect TEA gases as low as 1 ppm and exhibit excellent selectivity among six VOC gases. The efficient and sensitive sensing performance of ZnO/In_2_O_3_ is attributed to its large specific surface area, rich pore structure, and numerous active sites. This sensing platform that can detect biphasic pollutants has excellent prospects and good practicality.

## Data Availability

Data are contained within the article.
